# HIV Testing Among Muslim Women in the United States: Results of a National Sample Study

**DOI:** 10.1089/heq.2020.0041

**Published:** 2021-01-19

**Authors:** Kristine R. Hearld, Danielle Wu, Henna Budhwani

**Affiliations:** ^1^ Department of Health Services Administration, University of Alabama at Birmingham, Birmingham, Alabama, USA.; ^2^ Department of Nutrition, College of Human Ecology, Cornell University, Ithica, New York, USA..; ^3^ Department of Health Care Organization and Policy, University of Alabama at Birmingham, Birmingham, Alabama, USA.

**Keywords:** women's health, Islam, HIV testing, health

## Abstract

**Purpose:** More than one million Americans are living with human immunodeficiency virus (HIV), and less than half of Americans have ever accepted an HIV test. There are no national HIV testing estimates for Muslim Americans, an underserved and often stigmatized population. Considering the lack of HIV testing estimates for this population, we conducted an exploratory study on HIV testing and potential associates in American Muslim women from across the United States.

**Methods:** We applied logistic regression models to examine the Muslim Women's Health Project data, collected in 2015 (*N*=218).

**Results:** Health care engagement and intimate partner violence were significantly associated with having been tested for HIV. Respondents using contraceptives received an influenza vaccination, and received an abnormal pap test had more than two times higher odds of having been tested for HIV (odds ratio [OR]=2.56, OR=2.43, OR=2.93, respectively; *p*<0.05 all). Having been sexually abused was associated with more than two times higher odds of having been tested for HIV (OR=2.49; *p*<0.05).

**Conclusion:** Respondents reported higher rates of HIV testing as compared with the general public, signaling HIV knowledge, engagement in preventative health care, and possibly HIV risk. Scholars and practitioners should not assume that Muslim patients are at low risk for HIV and do not engage in HIV-risk behaviors. Thus, assumptions about Muslims women's willingness to accept HIV testing should be further examined to elucidate HIV risk among this population.

## Introduction

More than one million Americans are living with human immunodeficiency virus (HIV), and less than half of Americans have accepted an HIV test.^1,2^ There are no national HIV testing estimates for Muslim Americans, an underserved and often stigmatized population.^1–3^ Although Islamic doctrine condemns sexual risk behaviors, potentially reducing Muslim populations' rates of HIV and HIV testing, these assumptions are unsubstantiated. There is currently a lack of empirical evidence that confirms or refutes these beliefs.^4,5^ Considering the dearth of information on HIV testing among American Muslims, we analyzed data from 2015 Muslim Women's Health Project to assess potential associates of HIV testing that could be informative to public health interventionists and clinical practitioners engaged in HIV risk reduction.

### HIV among people of color and immigrants

Although national estimates of HIV-related behaviors in Muslim Americans are nearly nonexistent, some HIV research has been conducted that examines the behaviors of Muslim majority ethnicities (e.g., Arab Americans, migrants from Muslim majority countries) at the municipality or city level. For example, in Minnesota, two HIV testing studies conducted with Somalian immigrants found that this population demonstrated a lack of HIV knowledge and low rates of HIV testing.^5,6^ In a survey of Sudanese immigrants in Nebraska, researchers again found a notable lack of HIV knowledge.^7^ There is so little information on HIV-related outcomes in Muslim Americans, that the Arab Community Center for Economic and Social Services (ACCESS) lobbied the Michigan Department of Community Health to include an Arab ethnicity category to their HIV case reporting system.^8^ Thereafter ACCESS was able to identify people living with HIV of Arab descent and conduct focus groups among Arab men who have sex with men (MSM); Arab MSM participants disclosed their unwillingness to receive an HIV test for fear of community and family backlash (stigma).^8^ Respondents believed that only having sex with other Arab men would eliminate HIV risk.^8^ A related study found that stigma was a strong barrier to HIV testing, as well as to accurately reporting personal demographics (e.g., Arab, Muslim) in health care setting.^9^

Since Muslims are often racial and ethnic minorities, and some Muslim Americans identify as first-generation immigrants, HIV testing considerations related to minority and immigrant status may be applicable to American Muslim women. In the United States, HIV disproportionately affects minority populations with the largest percentage of undiagnosed infections clustered in racial and ethnic groups.^1^ Black and Latino populations consistently make up the largest proportion of new HIV infections.^1^ People of color face higher rates of poverty that can create barriers to care.^10^ Limited access to quality health care and low levels of HIV knowledge can indirectly and directly impact the ability of individuals to seek health care.^11,12^ For example, a survey of Latina women found that the low perception of personal risk was often cited reason for not getting tested.^13^ For undocumented immigrants, there is also a fear that seeking care could lead to deportation.^14^

### Women's health and preventative health

Globally, women account for half of HIV infections.^15^ Gender-based violence (GBV) and intimate partner violence (IPV) are associated with higher HIV risk; in our prior study, we have shown that American Muslim women have experienced high rates of stigma and abuse, which—by this logic—may put them at greater risk for HIV.^3^ In a study of women living with HIV, 90% of respondents reported being married and monogamous at the time they were infected with HIV, indicating that the previously stated assumption that Muslim women are at low risk due to cultural characteristics (e.g., few sexual partners, monogamy) may not be accurate.^15–18^ Marital conflicts, cultural tolerance of GBV, and fear of being socially ostracized influence women into being less likely to test for HIV.^19^

Related to Muslim women's engagement with the health care system, research has shown that this population utilizes health care less than other groups.^20–22^ A key factor related to avoidance of care seeking is the lack of gender-matched clinicians to provide care; it is common for American Muslim women to request a female physician or health worker.^23^ Because of this unaccommodated preference, as well as cultural considerations such as immigrating from countries that do not emphasize preventative health, prior research has shown lower rates of vaccination, use of contraceptives, and women's health screenings.^20–23^ Considering these characteristics, the Andersen Model of Health Services provides a meaning framework to conceptualize health care utilization among American Muslim women.^24–26^

### Andersen model of health services

This model offers a conceptual framework that elucidates personal and structural factors that influence (enable and constrain) health care utilization, particularly among subpopulations. The Anderson model suggests that health care engagement is affected by predisposing factors, enabling conditions, and medical need.^24–26^ Predisposing factors include race, age, and health beliefs, all of which are influenced by the Muslim identity. Enabling conditions may be social support, legal immigrant status in the United States, or even the availability to female providers in the community. For Muslim American women need encompasses actual (one is ill with symptoms) and perceived (perception that preventative care or HIV testing is warranted) need for care. In our study, we assess the effects of predisposing factors such as religious sect and age and enabling conditions, including prior engagement with health care services.

### Scientific rationale

Considering the scientific value of new knowledge discovery particularly related to HIV prevention, the dearth of data on hard-to-reach American subpopulations, and limited evidence about HIV testing and HIV risk in Muslim populations in the United States, we conducted this exploratory study.

## Methods

### Study design and participants

The Muslim Women's Health Project was conducted between September 2015 and December 2015 with the goal of collecting self-reported exploratory data on a range of health behaviors and outcomes from American Muslim women. Respondents recruitment took place through online social networks, e-mail requests, and postings made to digital Muslim communities. Potential respondents were asked to go to an online website to learn more about the study or to complete the study. Potential respondents were asked to participate in this anonymous survey of American Muslim women's experiences and to share the survey with other American Muslim women in their social networks. Respondents were not compensated for their participation. Questions were presented in a standard order, and answers were not required to progress through the survey, thus allowing respondents to skip any question.^3^ The respondent was not provided definitions for terms. Online surveys have been known to have limitations, such as sampling bias, but a benefit of online surveys is the ability to access difficult-to-reach populations.^27^ Owing to the employment of snowball sampling without unique personal identifiers, it was not possible to achieve geographic representation.

The primary outcomes for this substudy is HIV testing. Respondents were recruited through online social networks, e-mail requests, and postings made to online Muslim communities. Women who self-identified as Muslim, were at least 18 years old, and were current residents of the United States were eligible (*N*=218). The Muslim Women's Health Project was funded by the University of Alabama at Birmingham (UAB) School of Public Health, and the UAB Institutional Review Board provided ethical approval, #X150413001. This analysis was limited to respondents who reported being sexually active (*N*=218).

### Measures

Our primary outcome was HIV testing; respondents were asked if they had ever been tested for HIV. Respondents who reported that they been tested were recorded as 1 and those who had not were recorded as 0. Sociodemographics were assessed with five variables: age, marital status, education, income, and nativity. Age was measured as a continuous variable. Marital status was measured as a binary variable; married respondents were coded as 1 and not married respondents were coded as 0. Education was operationalized as a dummy variable, recorded as 1 if the respondent was a college graduate and 0 if not a college graduate. Income was included as a binary variable, coded as 1 if the respondent's household income was >$100,000/year and 0 if <$100,000/year. Finally, nativity was assessed as a binary variable, coded as 1 if the respondent was born in the United States and 0 if the respondent was born abroad.

Religion was included in two ways, through sect and frequency of worship attendance. Respondents were asked for their sect; response categories were Sunni, Shia, and general Islam (referent). General Islam was included for those who identified as culturally Muslims but did not align themselves with religious identities of Sunni or Shia. Frequency of worship attendance was measured with a dichotomously coded variable with 1 if the respondent attended worship once a week or more and 0 if the respondent attended worship less than once a week.

We included four health care engagement variables: use of contraception (any method), receipt of an influenza vaccination within the past 12 months, ever received an abnormal Papanicolaou test (commonly referred to as pap smears) result, and health insurance status. Respondents reporting “yes” to use of contraception were coded as 1, whereas respondents who answered “no” were coded as 0. Similarly, respondents were asked if they had received influenza vaccination within the past 12 months. Respondents who answered yes were coded as 1, whereas responses of no were coded as 0. Respondents were asked if they had ever received an abnormal pap test result. Respondents who had ever received an abnormal pap test result were coded as 1, whereas respondents who had never received a pap test were coded as 0. Health insurance status was categorized into three categories: (1) private health insurance, (2) public health insurance (such as Medicaid and Medicare), and (3) uninsured.

Finally, we included two variables reflecting GBV, and IPV. GBV was assessed with a question asking if respondents ever experienced sexual abuse by anyone. Respondents who answered yes were coded as 1, whereas responses of no were coded as 0. Similarly, respondents were asked if they had ever experienced physical abuse by a partner. Respondents who answered yes were coded as 1, whereas responses of no were coded as 0. Owing to sensitivity around disclosing experience with physical and sexual abuse, particularly in such an understudied minority population, multiple questions on physical and sexual abuse were not asked nor were definitions of physical abuse and sexual abuse provided.^3^

### Analytic strategy

Logistic regression models examine the relationship between HIV testing, sociodemographic characteristics, religiosity, and engagement with health care. Univariate statistics are displayed in Table 1 and multivariate results in Table 2. All variables were included in the multivariate models and estimated with Stata 15.0.

**Table 1.** Univariate Statistics Describing Characteristics of Muslim Women in the Study Sample (*N*=218)

	Muslim women
	M (SD)/*N* (%)
HIV test	
Receipt of HIV test (*N*/%)	100/45.87
Sociodemographic characteristics	
Age (M/SD)	35.0/10.87
Marital status	
Currently married (*N*/%)	148/67.89
Not married (*N*/%)	70/32.11
Education	
Less than college graduate (*N*/%)	31/14.22
College graduate (*N*/%)	187/85.78
Income	
<$100,000/year	112/51.38
>$100,000/year	106/48.62
Nativity	
U.S. born (*N*/%)	79/36.24
Foreign born (*N*/%)	139/63.76
Religious characteristics	
General Islam (*N*/%)	33/15.14
Shia (*N*/%)	109/50.00
Sunni (*N*/%)	76/34.86
Worship attendance	
Once a week or more (*N*/%)	56/25.69
Less than once a week (*N*/%)	162/74.31
Health care engagement	
Contraception use (*N*/%)	172/78.90
Influenza vaccine in past 12 months (*N*/%)	63/28.90
Abnormal pap test (*N*/%)	28/12.84
Health insurance status	
Uninsured (*N*/%)	11/5.05
Public insurance (*N*/%)	22/10.09
Private insurance (*N*/%)	183/83.94
Intimate partner violence and gender-based violence	
Sexual abuse	45/20.64
Physical abuse by intimate partner	32/14.68

SD, standard deviation.

**Table 2.** Multivariate Logistic Regression Predicting Associates of HIV Testing Among Muslim Women in the United States, *N*=218

	HIV test, OR (95% CI)
Sociodemographic characteristics	
Age	0.98 (0.95–1.01)
Marital status	
Currently married	1.19 (0.58–2.43)
Not married	Referent
Education	
Less than college graduate	0.72 (0.29–1.79)
College graduate	Referent
Income	
<$100,000/year	Referent
>$100,000/year	0.92 (0.48–1.77)
Nativity	
U.S. born	1.85 (0.93–3.68)
Foreign born	Referent
Religious characteristics	
General Islam	Referent
Shia	0.89 (0.357–2.200)
Sunni	0.559 (0.22–1.43)
Worship attendance	
Once a week or more	0.94 (0.45–1.93)
Less than once a week	Referent
Health care engagement	
Contraception use	2.63 (1.18–5.90)^*^
Influenza vaccine in past 12 months	2.32 (1.19–4.52)^*^
Abnormal pap test	2.93 (1.14–7.55)^*^
Health insurance	
Uninsured	Referent
Public insurance (Medicaid, Medicare)	0.32 (0.06–1.75)
Private insurance	1.17 (0.31–4.45)
Intimate partner violence and gender-based violence	
Sexual abuse	2.49 (1.11–5.59)^*^
Physical abuse by intimate partner	0.80 (0.40–1.59)

^*^ *p*<0.05; ^**^p<0.01; ^***^p<0.001.

CI, confidence interval; OR, odds ratio.

## Results

We present the sample characteristics of American Muslim women in Table 1 (*N*=218). Almost half of the respondents had received an HIV test at some point in their lives (45.9%). The mean age of respondents was 35.0 years and ranged from 18.9 to 68.7 years. Educational attainment was high with 85.8% reporting having a college degree. Approximately two-thirds of the sample reported being currently married (67.9%) and 36.2% were born in the United States. Almost half of respondents reported a household income of >$100,000 a year (48.6%). Half of the sample identified as Shia (50.0%), 34.9% identified as Sunni, and 15.1% identified as general Islam. One quarter of respondents reported attending worship services at least once a week (25.7%). Almost 80% of respondents reported use of contraception (78.9%); 28.9% reported having received an influenza vaccine in the past 12 months; 12.8% reported having received an abnormal pap test. Eighty-four percent of the respondents had private insurance (83.9%), 10.1% had public insurance, and 5.1% were uninsured. One in five women in the sample had experienced sexual abuse (20.6%) and 14.7% had experienced physical abuse by an intimate partner.

In Table 2, we report results from the logistic regression analysis examining the correlates of HIV testing. Only engagement with health care variables and IPV were significantly associated with having been tested for HIV. Respondents using contraception had more than two times higher odds of having been tested for HIV relative to respondents who did not use contraception (odds ratio [OR]=2.563, confidence interval [CI]=1.18–5.90, *p*<0.05). Respondents who had been vaccinated against influenza in the prior 12 months had more than two times higher odds of having been tested for HIV (OR=2.32, CI=1.19–4.52, *p*<0.05) compared with their peers who had not accepted an influenza vaccine in the prior year. Respondents who received an abnormal pap test had nearly three times higher odds of having been tested for HIV compared with respondents who never received an abnormal pap test result (OR=2.93, CI=1.14–7.55, *p*<0.05). Finally, respondents who reported having been sexually abused were associated with more than two times higher odds of having been tested for HIV (OR=2.49, CI=1.11–5.59, *p*<0.05).

## Discussion

The results of our study provide a preliminary estimate of HIV testing in our sample of Muslim women residing in the United States; about 48% (47.9%) reported having accepted an HIV test. This is notably higher than the 2019 prevalence rate of HIV testing in the general American public as reported by the Centers for Disease Control and Prevention (38%).^2^ Considering the strong push across medicine and public health to reach the 2020 HIV 90-90-90 targets, specifically, 90% of all people living with HIV will know their HIV status (HIV testing); 90% of all people with diagnosed HIV infection will receive sustained antiretroviral therapy, and 90% of all people receiving antiretroviral therapy will have viral suppression, scholars and practitioners alike would benefit by understanding structural forces and personal characteristics that may be predictive or restrictive of HIV testing.^28^ Respondents born in the United States were more likely to have been tested for HIV relative to those born abroad. Although such a finding may seem intuitive—those born in the United States are likely to be more acculturated and have higher levels of agency—it does not take into consideration that HIV testing was a requisite for migration into the United States up until less than a decade ago, and that nearly 60% of Muslims in the United States were born abroad.^14,29–31^ Considering this, the notable difference in HIV testing rates among American-born Muslim women and their immigrant peers (even after controlling for age) warrants further thoughtful examination that could lead to findings that are meaningful for HIV-testing efforts in similar diverse communities.

All health care engagement variables were significant predictors of HIV testing, indicating that perhaps ongoing and routine engagement in health care whether for nonreproductive health services (influenza vaccination) or for related health services (pap test and contraception) are pathways to promoting HIV testing; this provides some support for the value of expanding the patient-centered medical home model that routinely engages patients along the life course as well as the continuum of care.

### Limitations

Our sample was relatively small compared with the entire population of Muslim women in the United States. Selection bias is a concern; respondents were more educated and wealthier than Americans in general, and likely compared with all American Muslims. Online surveys have notable benefits, including enabling researchers to reach stigmatized, hard-to-reach populations, but there are also limitations such as the inability to reach disenfranchised subgroups, including women who lack English fluency and women with limited internet access. Because of limitations related to this modality (online surveys), it is likely that we were unable to reach many study eligible Muslim women who were not online. Social desirability bias is a known limitation of behavioral studies within which self-reported data are collected.

## Conclusion

American Muslim women are an understudied hard-to-reach population that experience high levels of stigma and violence, despite our sample's high levels of education and income. Yet, the women in our sample reported significantly higher rates of HIV testing as compared with the general public, which signals knowledge about HIV, HIV risk, and potentially engagement in behaviors that increase the likelihood of HIV exposure. These extrapolations indicate that the unsubstantiated assumptions mentioned earlier in this article are potentially incorrect. Therefore, scholars and practitioners should not assume that Muslim patients are at low risk for HIV and do not engage in HIV-risk behaviors. Further research with this population that evaluates the mechanisms that led to this sample's improved rates of HIV testing could provide extensible insights to inform the development interventions designed to promote HIV testing in subpopulations of women across the United States.

## Abbreviations Used

ACCESS: Arab Community Center for Economic and Social Services
CI: confidence interval
GBV: gender-based violence
IPV: intimate partner violence
MSM: men who have sex with men
OR: odds ratio
SD: standard deviation
UAB: University of Alabama at Birmingham
